# Blockade of High-Fat Diet Proteomic Phenotypes Using Exercise as Prevention or Treatment

**DOI:** 10.1074/mcp.TIR120.002343

**Published:** 2020-12-19

**Authors:** Sergio F. Martinez-Huenchullan, Isaac Shipsey, Luke Hatchwell, Danqing Min, Stephen M. Twigg, Mark Larance

**Affiliations:** 1 Faculty of Science, Charles Perkins Centre, School of Life and Environmental Sciences, University of Sydney, New South Wales, Australia; 2 Faculty of Medicine and Health, Central Clinical School, University of Sydney, New South Wales, Australia; 3 Faculty of Medicine, School of Physical Therapy, Austral University of Chile, Valdivia, Chile; 4 Department of Endocrinology, Royal Prince Alfred Hospital, New South Wales, Australia

**Keywords:** exercise, proteomics, high-fat diet, plasma, APOA4, apolipoprotein A-IV, APOC2, apolipoprotein C-II, APOE, apolipoprotein E, CFD, complement factor D, Ctrl, control diet, DIA, data-independent acquisition, END, endurance training, FDR, false discovery rate, HFD, high-fat diet, HIIT, high-intensity interval training, Hp, haptoglobin, LIFR, leukemia inhibitory factor receptor, MRC, maximal running capacity, NAFLD, nonalcoholic fatty liver disease, PUFA, polyunsaturated fatty acid, SFA, saturated fatty acid

## Abstract

The increasing consumption of high-fat foods combined with a lack of exercise is a major contributor to the burden of obesity in humans. Aerobic exercise such as running is known to provide metabolic benefits, but how the overconsumption of a high-fat diet (HFD) and exercise interact is not well characterized at the molecular level. Here, we examined the plasma proteome in mice for the effects of aerobic exercise as both a treatment and as a preventative regimen for animals on either a HFD or a healthy control diet. This analysis detected large changes in the plasma proteome induced by the HFD, such as increased abundance of SERPINA7, ALDOB, and downregulation of SERPINA1E and complement factor D (CFD; adipsin). Some of these changes were significantly reverted using exercise as a preventative measure but not as a treatment regimen. To determine if either the intensity or duration of exercise influenced the outcome, we compared high-intensity interval training and endurance running. Endurance running slightly outperformed high-intensity interval training exercise, but overall, both provided similar reversion in abundance of plasma proteins modulated by the HFD, including SERPINA7, apolipoprotein E, SERPINA1E, and CFD. Finally, we compared the changes induced by overconsumption of a HFD with previous data from mice fed on an isocaloric high-saturated fatty acid or polyunsaturated fatty acid diet. This identified several common changes, including not only increased apolipoprotein C-II and apolipoprotein E but also highlighted changes specific for overconsumption of a HFD (fructose-bisphosphate aldolase B, SERPINA7, and CFD), saturated fatty acid–based diets (SERPINA1E), or polyunsaturated fatty acid–based diets (haptoglobin). Together, these data highlight the importance of early intervention with exercise to revert HFD-induced phenotypes and suggest some of the molecular mechanisms leading to the changes in the plasma proteome generated by HFD consumption. Web-based interactive visualizations are provided for this dataset (larancelab.com/hfd-exercise), which give insight into diet and exercise phenotypic interactions on the plasma proteome.

## Highlights

• Plasma proteomics reveals 30% of proteins change in response to a high-fat diet (HFD).
• Running exercise applied from the beginning of the HFD reverts some of these changes.
• High-intensity interval training and endurance training provide similar benefits.
• Some proteins were regulated by either HFD overconsumption or diet fat profile.

## Graphical Abstract

**Figure:**
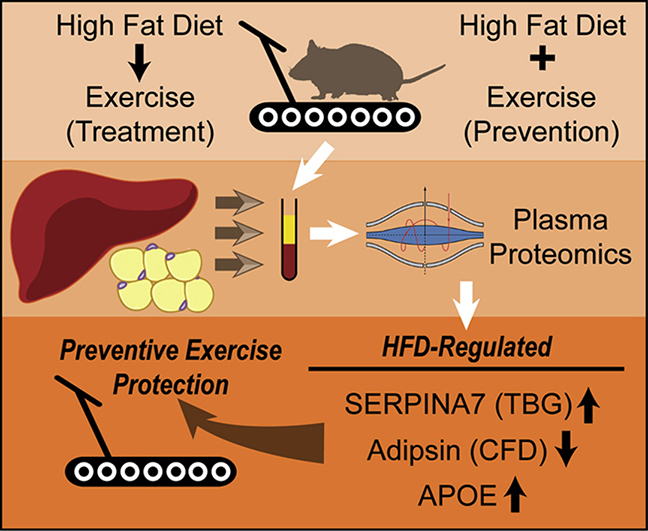

## In Brief

Exercise is known to provide benefits, but how a high-fat diet (HFD) and exercise interact is not well characterized. Here, we examined the plasma proteome in mice for the effects of exercise as both a treatment and a preventative regimen for animals on either a HFD or a healthy control diet. Many changes in the plasma proteome were induced by the HFD, and these were significantly reverted using exercise as a preventative measure but not as a treatment regimen.

Obesity is at epidemic levels in the developed world with many Western countries having >25% of their population being classified as obese (1). Obesity promotes organ dysfunction and changes in circulating measures of metabolic disease (2). For example, obesity is frequently associated with nonalcoholic fatty liver disease (NAFLD) and associated premature mortality, ultimately linked to cardiovascular disease, some cancers, and type 2 diabetes (1). Obesity is often modeled in animals such as rodents through the provision of high-fat diets (HFDs) *ad libitum*. This allows the voluntary consumption of excess calories because of both the high-energy density of the HFD and the increased appetite for high-fat food compared with normal chow diets (3). In mice, the *ad libitum* consumption of a HFD for 1 week is sufficient to induce liver insulin resistance (4). Usually more than 3 weeks of *ad libitum* HFD is enough to also induce muscle and adipose tissue insulin resistance (4), which are key pathways in the induction of type 2 diabetes. However, recent studies have shown that if mice are provided a HFD in an isocaloric manner through pair-feeding with chow-fed animals, the animals given the isocaloric HFD quickly adapt to the altered nutrient proportions and display improved metabolic phenotypes compared with the animals given chow (5). This suggests it is the excess consumption of calories and not just the high-fat content of the diet, which is responsible for the negative outcomes on metabolic health. This agrees with findings from studies of ketogenic diets that are very high in fat content but provide metabolic benefits (6).

Given the effects of obesity induced by the overconsumption of calories, strategies that aim to prevent and/or treat obesity-related dysfunction are of increasing interest. Exercise can attenuate adverse metabolic changes in a variety of disease contexts (7). Currently, the most effective exercise prescription (*e.g.*, intensity, duration, frequency) to use in the presence of obesity is unclear. To address this, a comparison between constant-moderate endurance (END) and high-intensity interval training (HIIT) protocols, in different metabolic conditions, has been informative and showed that both exercise regimens induced similar reductions in total body mass and waist circumference (8). However, HIIT required approximately 40% less training time commitment to promote those changes (8). Few studies have aimed to elucidate molecular pathways and mechanisms behind desirable changes induced by exercise, especially in a high-fat intake environment. Recently, Groussard *et al.* (9), when investigating effects of 10 weeks of END and HIIT, described tissue-specific effects in obese Zucker rats on oxidative stress modulators in white adipose tissue and skeletal muscle. Endurance exercise increased catalase and glutathione peroxidase activities in adipose tissue, whereas HIIT specifically increased glutathione peroxidase activity (9). These results are aligned with our research in high-fat fed mice, where 10 weeks of END *versus* HIIT exerted differential metabolic effects on liver, white adipose tissue, and quadriceps muscle, and END specifically reduced fibrotic markers in liver, whereas HIIT increased uncoupling protein 1 in adipose tissue (10), highlighting that these programs may have specific metabolic effects in an obesity context. While most relevant literature indicates that exercise in any form exerts metabolic benefits during obesity, it is unclear whether preventative exercise while obesity develops, or exercise treatment in an individual who is already obese, has similar effects or if certain exercise prescriptions have metabolic advantages (11).

In this study, we investigated the plasma proteome in mice for the effects of two aerobic exercise approaches (END and HIIT) that were provided in either a treatment regimen or a preventative regimen, for animals provided food *ad libitum* that was either a HFD or a healthy control diet (10, 12). We identified many significant changes to the plasma proteome in response to the HFD and showed that only a small proportion of these could be blocked by exercise regimes. We showed that a preventative exercise regimen is required to have significant effects and that both endurance and HIIT exercise modes provided similar benefits. By comparing with a previous dataset, we could identify HFD-induced plasma protein abundance changes that were specific to the overconsumption of a HFD in our model. Furthermore, we could use this comparison to delineate the required fatty acid saturation needed to generate these changes. These data provide a comprehensive overview of the plasma proteomic phenotypes generated by HFD exposure and its interaction with several aerobic exercise regimens, which are provided as an online resource (larancelab.com/hfd-exercise).

## Experimental Procedures

### Chemicals and Reagents

Acetonitrile (Optima grade), acetone, water (Optima grade), ammonia, formic acid, and isopropanol (Optima grade) were from Thermo Fisher Scientific. Ethylacetate LC–MS grade was from Millipore. 3-(4-Carboxybenzoyl)quinoline-2-carboxaldehyde reagent was from Applied Bioprobes. Proteomics-grade trypsin (catalog number: T6567) and all other reagents were from Sigma–Aldrich.

### Animal Details

The samples used in this study are derived from previous physiological studies of mice (10, 12). Briefly, male C57BL/6J mice were purchased from the Animal Resources Centre (Perth, Australia) and housed in individually ventilated cages with corn cob bedding (Bed-o'Cobs 1/8”, Andersons), in groups of *n* = 4 mice per cage, for at least 1 week before entering experimental models. Mice were maintained in a temperature-controlled room (22 °C ± 1 °C) with a 12-h light/dark cycle (0600/1800 h) with *ad libitum* access to water and food, which was either a standard chow diet (12% calories derived from fat, 23% calories from protein, and 65% calories from carbohydrates; Specialty Feeds, Glen Forrest) or a HFD made inhouse according to the formula from Research Diets, Inc (catalog no: D12451; 45% calories derived from fat, 20% calories from protein, and 35% calories from carbohydrates). All experiments were carried out with the approval of the University of Sydney Animal Ethics Committee (2015/816), following the National Health and Medical Research Council of Australia and Animal Research: Reporting of *In Vivo* Experiments guidelines. Mice from different cages were used to negate cage-specific effects.

In the first study (exercise treatment) (10), mice at 10 weeks of age were randomly assigned into either chow or HFD groups (*n* = 36 per group). After 10 weeks of diet without exercise, the mice in each dietary group were then randomized into one of three exercise groups: no exercise training (none), endurance treadmill running, or HIIT treadmill running (*n* = 12 per group), which continued for 10 weeks.

In the second study (preventative exercise) (12), mice at 10 weeks of age were randomly assigned into either chow or HFD groups (*n* = 36 per group) and were then immediately randomized to one of three exercise groups: no exercise training (none), endurance treadmill running, or HIIT treadmill running (*n* = 12 per group), which continued for 10 weeks.

### Treadmill-Based Exercise Regimes

Mice were acclimatized to the treadmill for 1 week, and then a maximal running capacity (MRC) test was performed for each animal (10, 12). The exercise intensity of the two different training programs (endurance or HIIT) was calculated using the animal's MRC. Importantly, the exercise programs were designed to be comparable in terms of exercise volume and distance covered per session. For endurance exercise, a constant running session of 70% MRC (17 m/min) for 40 min was used, whereas for HIIT, eight bouts (2.5 min each) at 90% of the MRC (22 m/min) intercalated by eight active rest periods (2.5 min each) at 50% of the MRC (12 m/min) (40 min total per session), were used. Each training program was performed in the morning, three times per week for 10 weeks. Nonexercised animals were not exposed to additional exercise.

### Tissue Collection

Mice were euthanized after an overnight period of feeding by terminal anesthesia with isoflurane (3%) in oxygen, starting at 0900 h with all animals euthanized by 1100 h. Plasma was collected *via* cardiac puncture into tubes precoated with 0.5 M EDTA at 10% recovered blood volume. Whole blood was kept on ice and then spun at 1500×*g* for 15 min at 4 °C, with collected plasma snap frozen in liquid nitrogen before being stored at −80 °C.

### Plasma Sample Preparation Using Styrenedivinylbenzene–Reverse Phase Sulfonate StageTips

Plasma sample preparation was performed as described previously (13). Briefly, 1 μl of plasma (70 μg protein) was added to 24 μl of sodium deoxycholate buffer (1% sodium deoxycholate, 10 mM tris(2-carboxyethyl)phosphine, 40 mM chloroacetamide, and 100 mM Tris–HCl, pH 8.5) and heated to 95 °C for 10 min. Once cooled to room temperature, the sample was diluted 10-fold with water. LysC and trypsin were then added at a 1:100 ratio (μg/μg) and digested at 37 °C for 16 h. An equal volume (250 μl) of 99% ethylacetate/1% TFA was added to the digested peptides and vortexed. Digested peptides were purified using styrenedivinylbenzene–reverse phase sulfonate StageTips and the Spin96 as described (13). Dried peptides were resuspended in 30 μl of 5% formic acid and stored at 4 °C until analyzed by LC–MS.

### LC–MS/MS and Analysis of Spectra

Using a Thermo Fisher RSLCnano ultrahigh performance liquid chromatography, peptides in 5% (vol/vol) formic acid (injection volume of 3 μl) were directly injected onto a 15 cm × 150 μm C18Aq (Dr Maisch; 1.9 μm) fused silica analytical column with a ∼10 μm pulled tip, coupled online to a nanospray electrospray ionization source. Peptides were resolved over gradient from 5% acetonitrile to 40% acetonitrile over 25 min with a flow rate of 1200 nl min^−1^ (capillary flow). Peptides were ionized by electrospray ionization at 2.3 kV. Tandem mass spectrometry analysis was carried out on a Q-Exactive HFX mass spectrometer (Thermo Fisher) using data-independent acquisition (DIA). The DIAs were performed as described previously using variable isolation widths for different m/z ranges (14). Stepped normalized collision energy of 25 ± 10% was used for all DIA spectral acquisitions.

Raw data were analyzed using the quantitative proteomics software Spectronaut Pulsar X (version 12.0.20491.11.25225 [Jocelyn]). DirectDIA analysis was used to identify peptides (15). The database supplied to the search engine for peptide identifications was the mouse UniProt database downloaded on July 15, 2019, containing 63,439 protein sequence entries. Enzyme specificity was set to semispecific N-ragged trypsin (C-terminal cleavage to Lys and Arg) with a maximum of two missed cleavages permitted. Deamidation of Asn and Gln, oxidation of Met, pyro-Glu (with peptide N-terminal Gln), and protein N-terminal acetylation were set as variable modifications. Carbamidomethyl on Cys was searched as a fixed modification. To recalibrate for retention drift and intensity assignment for peaks, the indexed retention time profiling workflow was used (16). For peak list generation, interference (MS1 and MS2) correction was enabled, removing fragments/isotopes from potential quantitation if there was a presence of interfering signals, while keeping a minimum of three for quantitation. Spectra were deisotoped based on RT apex distance and m/z spacing, and demultiplexing was not required. Each observed fragment ion could only be assigned to a single precursor peak list (15). The false discovery rate (FDR) was set to 1% using a target-decoy approach. Spectronaut generated a custom mass tolerance and retention time tolerance for each precursor ion and its product ions. The threshold for accepting a precursor was set at a Q value <0.01, and each precursor must have >3 fragment ions. All other settings were factory default.

### Experimental Design and Statistical Rationale

The number of animals used per treatment group was established from previous studies in mouse muscle high–molecular weight adiponectin (12). We have used the values for high–molecular weight adiponectin as pilot data to provide suitable values for power calculation. At 90% power, with a worst case true difference in means of 200, a representative standard deviation of 130, probability of type I error of 0.05, and using a one-way ANOVA test, the observations that are required are *n* = 10, and we therefore used 10 animals per treatment group. For all datasets, statistical analyses were performed using R (version 3.4.3), and processed data were plotted using Tableau (version 2019.2), where outliers beyond 1.5 times the interquartile range may have been excluded from plots to aid visualization. Fold changes for protein abundance were calculated using the median in each group. Statistical significance for changes induced by diet condition (chow *versus* HFD), exercise (none *versus* endurance *versus* HIIT), or the interaction between these was calculated using a two-way ANOVA. In addition, the HFD group only was analyzed using a one-way ANOVA to examine the effect of exercise in this group alone. The resulting *p* values from both tests were adjusted to control for multiple testing using the Benjamini–Hochberg correction. Significance was set at *p* < 0.05, corresponding to a FDR in the ANOVA of 5%.

## Results

### Plasma Proteome Analysis of HFD-Fed Animals With Exercise as Treatment

**Fig. 1:**
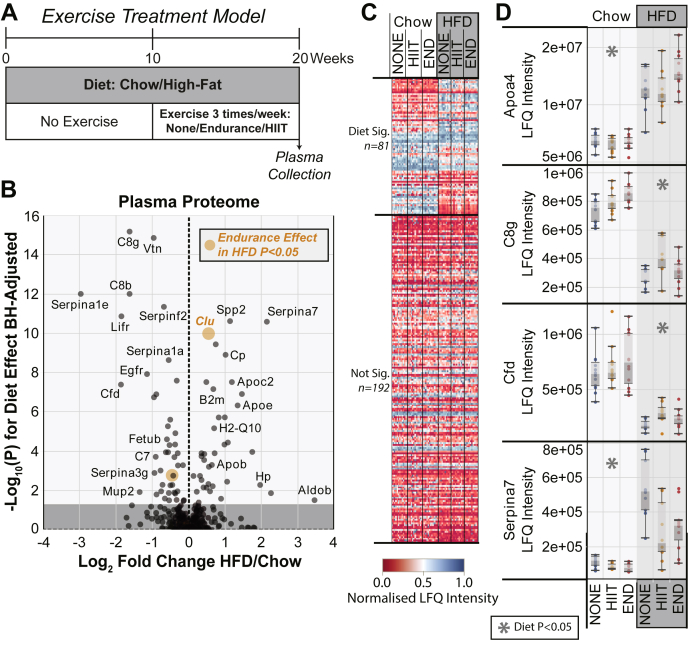
**Plasma proteomics of high-fat diet (HFD) and exercise treatment interaction.** Mice were provided either a chow diet or a HFD, *ad libitum* for 10 weeks. After this time, animals from each diet group were randomly assigned to exercise treatments of none, endurance (END), or high-intensity interval training (HIIT). *A*, schematic of the diet and exercise schedule with the timing of blood plasma collection. *B*, volcano plot for all plasma proteins detected, with the log_2_ fold change (HFD/chow) plotted on the *x*-axis *versus* the Benjamini–Hochberg corrected −log_10_(P) for the diet effect from the two-way ANOVA shown on the *y*-axis. The *dark gray* region highlights nonsignificant *p* values (>0.05). The size and color of the points represent significance of any exercise effect within the HFD group alone as indicated by the legend. *C*, heat map of the plasma proteome analyzed by two-way ANOVA, in which *blue* indicates high protein abundance and *red* indicates low protein abundance. Each column represents an individual mouse. *D*, box and whisker plots for specific proteins of interest. Each point represents protein abundance from an individual mouse. *Asterisks* placed at the top of any plot represents statistical significance (*p* < 0.05 or 5% false discovery rate) across the treatment variables as indicated by the legend (*n* = 12 per group). LFQ, label-free quantification.

The plasma proteome contains a large range of circulating factors secreted from many organs/tissues of the body, particularly the liver. The most abundant proteins in plasma are enriched in factors mediating lipid metabolism and lipid transport, which are of particular interest in relation to HFD studies. For the analysis of the interaction between a HFD and an aerobic exercise, we started with plasma samples from a standard exercise treatment model described previously (10). In this model, C57BL/6J male mice were used, and the intervention was commenced at 10 weeks of age. Animals were fed either a HFD (45% kcal as fat) or normal chow (12% kcal as fat) for 10 weeks without any exercise treatment and were then switched to a combined HFD and exercise treatment regimen for the subsequent 10 weeks prior to plasma collection (Fig. 1*A*). The treatment consisted of no exercise training (none), treadmill-based HIIT, or treadmill-based END. Exercise bouts of 40 min each were repeated 3 times per week. Each treatment group had 8 to 12 animals. We characterized the blood plasma from each animal harvested in the *ad libitum* fed state with undepleted plasma proteomics (13) using trypsin digestion and LC–MS/MS analysis by DIA. This yielded the identification of 273 protein groups (supplemental Table S1) from a total of >5100 peptides (supplemental Tables S2 and S3) across the six animal groups on each diet (chow *versus* HFD) combined with an exercise treatment (none *versus* HIIT *versus* END).

To determine which proteins were significantly altered by either the diet or the exercise treatment, we applied a two-way ANOVA to each protein detected in this proteomic dataset. The *p* values derived from this test were corrected for multiple testing using the method of Benjamini–Hochberg. This analysis also allowed us to determine if there was any significant interaction between the diet variable and the exercise treatment variable. This analysis detected 82 proteins that had a *p* < 0.05 (<5% FDR) for a significant difference between the HFD and chow fed animals, irrespective of exercise (Fig. 1*B*). This constitutes ∼40% of the detected plasma proteome and shows the strong influence of the HFD on mammalian physiology. Comparing the different exercise regimens, we detected only four proteins that were significantly regulated by exercise treatment. Finally, there were no proteins that showed a significant interaction between the diet and exercise variables. Detailed analysis using a one-way ANOVA across the exercise treatment groups in only the HFD-fed animals showed that endurance exercise, but not HIIT, had a small but significant effect of reverting the HFD changes on 2 proteins: clusterin and transthyretin.

To provide an overview of the changes induced by the treatments across all animals, we plotted a heat map of the normalized label-free quantitation intensity for all proteins detected with proteins separated by significance grouping (Fig. 1*C*). This confirmed the consistency of response across each treatment group and the minor effect of exercise as a treatment for animals already exposed to a HFD for a 10-week period. Some of the proteins with the largest significant fold-change increase in response to the HFD were thyroxine-binding globulin (SERPINA7), apolipoprotein C-II (APOC2), haptoglobin (Hp), apolipoprotein E (APOE), apolipoprotein A-IV (APOA4), and fructose-bisphosphate aldolase B (ALDOB, liver isoform) (Fig. 1, *B* and *D*). Among the most significantly downregulated proteins were complement factor D (CFD—adipsin), the major mouse α1-antitrypsin isoform (SERPINA1E), complement component C8 β/γ chains (C8b/g), and the soluble form of the leukemia inhibitory factor receptor (LIFR) (Fig. 1, *B* and *D*). Most proteins significantly altered by the HFD are known to be secreted from the liver (17), highlighting the importance of this tissue in the response to HFD and the liver's role in producing most of the abundant plasma proteins. Exceptions to this would include CFD and APOA4, which are mainly adipose- and intestine-derived proteins, respectively. A small subset of 10 proteins significantly altered by the HFD are not known to be secreted including ALBOB, PSMA2, SNX13, HSP1A1, and ERN1, which may have been released into the circulation because of liver cell death. Finally, in this exercise-treatment cohort, only two proteins, transthyretin and clusterin, had small but statistically significant exercise-induced effects to correct the HFD-induced changes. Importantly, the exercise effects on these two proteins were only significant after endurance exercise but not HIIT.

### Plasma Proteome Analysis of HFD-Fed Animals With Exercise-Based Prevention

**Fig. 2:**
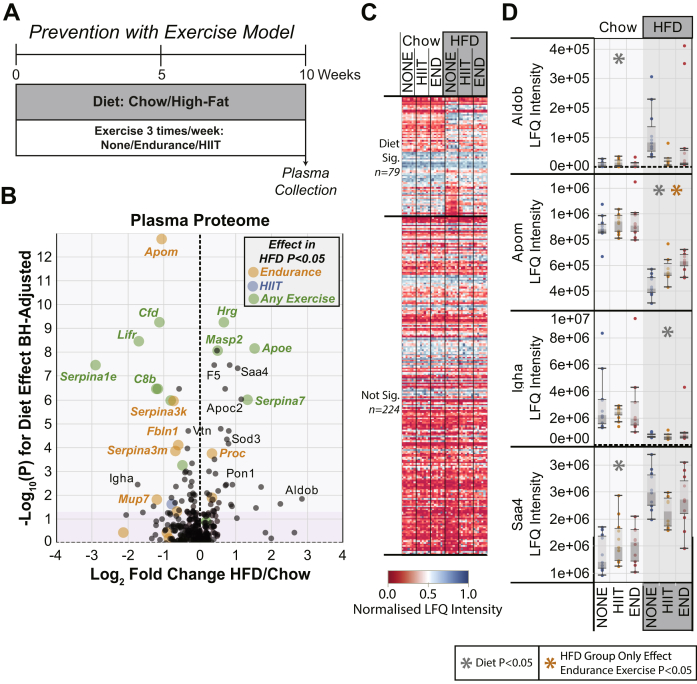
**Plasma proteomics of high-fat diet (HFD) and preventative exercise interaction.** Mice were provided either a chow diet, or a HFD, *ad libitum* for 10 weeks and simultaneously from each diet group were assigned to exercise treatments of none, endurance (END), or high-intensity interval training (HIIT). *A*, schematic of the diet and exercise schedule with the timing of blood plasma collection. *B*, volcano plot for all plasma proteins detected, with the log_2_ fold change (HFD/chow) plotted on the *x*-axis *versus* the Benjamini–Hochberg corrected −log_10_(P) for the diet effect from the two-way ANOVA shown on the *y*-axis. The *dark gray* region highlights nonsignificant *p* values (>0.05). The size and color of the points represent significance of any exercise effect within the HFD group alone as indicated by the legend. *C*, heat map of the plasma proteome analyzed by two-way ANOVA, in which *blue* indicates high protein abundance and *red* indicates low protein abundance. Each column represents an individual mouse. *D*, box and whisker plots for specific proteins of interest. Each point represents protein abundance from an individual mouse. Asterisks placed at the top of any plot represent statistical significance (*p* < 0.05 or 5% false discovery rate) across the treatment variables as indicated by the legend (*n* = 12 per group). LFQ, label-free quantification.

We next wanted to analyze the effect of exercise as a preventative measure using plasma samples from a model previously described (12), where the animals started the HFD and the exercise regimen at the same time and continued for 10 weeks in total (Fig. 2*A*). We hypothesized that starting the exercise intervention earlier should have a much bigger effect to revert HFD-induced plasma proteome changes. Again, we used the same two-way ANOVA as for the treatment model. This showed that 79 proteins had a significant difference (<5% FDR) between the HFD animals and the chow-fed animals, irrespective of exercise (Fig. 2*B*). However, we now observed 24 proteins that were significantly regulated by exercise treatment and 18 proteins that showed a significant interaction between the diet variable and the exercise variable (Fig. 2*B*). Detailed analysis using a one-way ANOVA across the exercise treatment groups in the HFD-fed animals only shows that endurance exercise alone had a significant effect on 11 proteins, HIIT alone had an effect on 1 protein, and 12 proteins were significantly affected by both endurance and HIIT. The heat map plot of the plasma proteome across the animal groups shows several subsets of proteins altered by the HFD that are modified similarly by both preventative exercise regimens (Fig. 2*C*). The proteins with the largest significant fold-change increase in response to the HFD in this prevention model were very similar to those observed in the treatment model and included ALDOB, APOE, APOC2, Hp, and serum amyloid A-4 (Fig. 2, *B* and *D*). The most significantly downregulated proteins also showed similarity, including LIFR, epidermal growth factor receptor, C8B/G, SERPINA1E, and CFD (adipsin) (Fig. 2*B*).

**Fig. 3:**
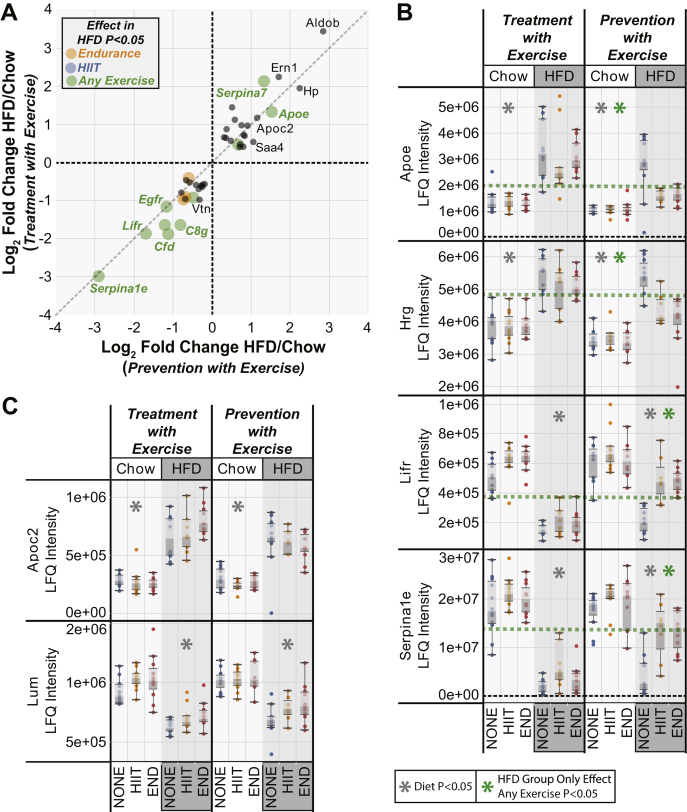
**Comparison of preventative exercise *versus* exercise treatment datasets for high-fat diet (HFD) response to exercise efficacy.***A*, proteins that were significantly regulated by the HFD in both the exercise prevention and treatment models are plotted. Each point shows the log_2_ fold change for the HFD/chow response in the treatment model (*y*-axis) and the prevention model (*x*-axis). The size and color of the points represent significance of any exercise effect in the prevention model for the HFD group alone as indicated in the legend. *B*, box and whisker plots for specific proteins of interest showing a differential response to exercise applied as either a treatment or a prevention model. *C*, box and whisker plots for specific proteins that did not significantly respond to exercise. Each point in *B*–*C* represents protein abundance from an individual mouse. Asterisks placed at the top of any plot represents statistical significance (*p* < 0.05 or 5% false discovery rate) across the treatment variables as indicated in the legend (*n* = 12 per group). HIIT, high-intensity interval training; LFQ, label-free quantification.

To directly compare the HFD response of the plasma proteome between the treatment and preventative exercise regimens, we generated a scatter plot for all those proteins that showed a significant effect of HFD in both the exercise treatment and prevention with exercise models. The fold change in both the prevention and treatment models was used as the plot *x*- and *y*-axes, respectively (Fig. 3*A*). Proteins displaying the same response to the HFD would align on a 45-degree line (Fig. 3*A*—*gray dashed line*). This plot showed that most proteins upregulated by the HFD had similar fold changes (HFD/chow) across the exercise model datasets. In contrast, proteins downregulated by the HFD in general showed a reduced fold change in the prevention model potentially associated with the decreased total exposure time to the HFD (10 *versus* 20 weeks). Proteins that were significantly upregulated by the HFD in both the preventative and treatment exercise model datasets were analyzed for pathway enrichment (supplemental Fig. S1*A*), which showed many of these proteins are involved in lipid metabolism and lipid transport and several are known to interact directly on lipoprotein particles such as high-density lipoprotein. Proteins that were significantly downregulated by the HFD in both the preventative and treatment exercise model datasets (supplemental Fig. S1*B*) showed enrichment in protease inhibitors, coagulation factors, and complement factors, with many of these also known to interact in protein complexes.

This comparison plot also highlights those proteins in the prevention model that showed a significant interaction between the HFD and exercise (Fig. 3*A*). Examination of box plots for some of these proteins highlights the differences in exercise-mediated reversion in response to the HFD (Fig. 3*B*). For example, in both the exercise treatment and prevention models, the APOE protein was induced ∼twofold by the HFD alone with no exercise. Exercise treatments of either HIIT or endurance running showed a mild reversion of APOE protein abundance to chow values in the treatment model, but the response in the prevention model was significantly larger. A similar profile was also seen for histidine-rich glycoprotein with the exercise response being even more marked in the prevention model. Conversely, both the soluble LIFR protein and SERPINA1E showed strong reversion of protein abundance with preventative exercise compared with the more than threefold decrease in response to a HFD in either the absence of exercise or in the treatment exercise model. However, most plasma proteins did not show any interaction between the HFD and preventative exercise, and two examples are the APOC2 protein and the lumican protein (Fig. 3*C*). Both these proteins showed similar responses to a HFD regardless of experimental model and exercise exposure.

### Comparison of HFD Response When Provided *ad Libitum Versus* an Isocaloric Diet Study

**Fig. 4:**
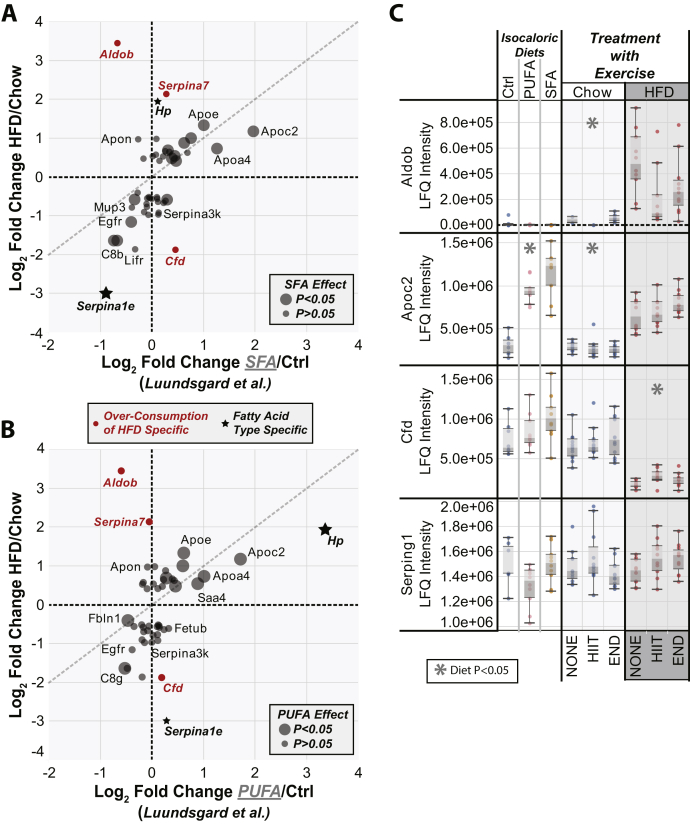
**Comparison of the plasma proteome response to a high-fat diet (HFD) between isocaloric and overconsumption diet regimens.***A*–*B*, proteins that were significantly regulated by the HFD in the exercise treatment model are plotted. The *y*-axis shows log_2_ fold change for the HFD/chow response in the treatment model. *A*, the *x*-axis shows the log_2_ fold change to a saturated fatty acid (SFA)–rich diet compared with a chow control diet (Ctrl), which were both provided in an isocaloric manner (pair fed) (5). *B*, the *x*-axis shows the log_2_ fold change to a polyunsaturated fatty acid (PUFA)–rich diet compared with a chow control diet (Ctrl), which were both provided in a isocaloric manner (pair fed) (5). For *A*–*B*, the size, shape, and color of the points represent significant differences between either isocaloric *versus* overconsumption HFD models (*red points*), or the SFA *versus* PUFA diets (*solid black stars*), as indicated in the legends. *C*, box and whisker plots for specific proteins of interest. Each point represents protein abundance from an individual mouse. Asterisks placed at the top of any plot represents statistical significance (*p* < 0.05 or 5% false discovery rate) across the treatment variables as indicated in the legend (*n* = 12 per group). LFQ, label-free quantification.

Mice provided with a HFD *ad libitum* (free access) will consume a similar mass of food to chow-fed animals, but given the significantly higher energy density of a HFD, those mice will gain weight rapidly compared with chow-fed animals, as seen for both of the models examined in this study (10, 12) (supplemental Fig. S2). We wanted to determine the differences in the plasma proteome response (HFD/chow) when the diets were provided either *ad libitum* (this study) or as an isocaloric pairing between HFD-fed and chow-fed animals (5). In the previous isocaloric pair-feeding study of mice with plasma proteome analysis data, two different HFDs were compared with a chow control diet (Ctrl), which were either high in saturated fatty acid (SFA) or high in polyunsaturated fatty acid (PUFA) (5). This allowed us to compare the effects of both diet overconsumption and diet composition on the plasma proteome. Again, we generated scatter plots comparing the fold changes (HFD/chow) between the study of Lundsgard *et al.* (5) and our treatment model data for all those proteins that showed a significant effect of a HFD in our dataset (Fig. 4).

Comparison of our data with either SFA or PUFA response showed many similar responses such as increased abundance of APOC2, APOE, and APOA4. Other common changes included decreased abundance of the soluble epidermal growth factor receptor and the complement protein 8 components (C8B/C8G). Therefore, these protein abundance changes are likely because of the consumption of food containing a high proportion of fat regardless of the amount of energy consumed. Several stand-out differences between the overconsumed *versus* isocaloric HFD were ALDOB, SERPINA7, and CFD (adipsin) (Fig. 4—*red points*). Both ALDOB and SERPINA7 were markedly increased in plasma from animals overconsuming the HFD, whereas the response in the isocaloric HFD animals was either very small (SERPINA7) or the inverse response (ALDOB). Another protein showing an inverse response was CFD (adipsin), which was significantly downregulated by overconsumption of the HFD but was slightly increased in abundance in the isocaloric HFD dataset.

There were several proteins whose response to fatty acid composition was distinct, including α1-antitrypsin (SERPINA1E) and Hp (Fig. 4—*black stars*). SERPINA1E showed significant downregulation in our overconsumption HFD (composed of a mixture of saturated and PUFAs/monounsaturated fatty acids) animals and the isocaloric SFA group (Fig. 4*A*). In contrast, SERPINA1E was not significantly changed in animals of the isocaloric PUFA group (Fig. 4*B*). Therefore, SERPINA1E abundance is likely controlled by SFA exposure regardless of amount consumed. Conversely Hp was upregulated in both our overconsumption HFD animals and the isocaloric PUFA group (Fig. 4*B*), but not the SFA group, suggesting Hp abundance is controlled by PUFA exposure regardless of the total high fat food consumed. Comparison of the raw data for selected proteins from each of these studies confirms these observations and highlights the dramatic change in ALDOB abundance in animals overconsuming the HFD (Fig. 4*C*).

## Discussion

In this study, we used plasma proteome analysis to characterize the interaction between HFD consumption and aerobic exercise in mice. Our goal was to identify circulating proteins that were modulated by the HFD and to determine if exercise applied as either a treatment, or a preventative measure, could rectify those HFD-induced changes. This study provides four key findings. First, animals fed a HFD showed marked changes in >30% of the detected plasma proteins compared with chow-fed animals. Second, exercise applied from the beginning of the HFD as a preventative measure was able to revert the changes for <20% of these proteins. In contrast, the use of exercise as a treatment after an extended period on a HFD only showed minor effects. Third, the type of aerobic exercise applied did not make a significant difference, with both HIIT and endurance training providing similar benefits for most proteins with few exceptions. Finally, a comparison of our *ad libitum* HFD response to a previous plasma proteome dataset in which a HFD was provided in an isocaloric regimen identified several proteins that were differentially regulated. This comparison also allowed us to identify plasma proteins whose response was triggered by either SFAs or PUFAs in the diet. Together, these findings show that the interaction of dietary lipid consumption and aerobic exercise, when provided as early as possible, can revert significant physiological changes associated with these mouse models of human disease. Web-based visualizations for this dataset are available to the scientific community (larancelab.com/hfd-exercise), which allow interrogation of diet and exercise phenotypic interactions on the mouse plasma proteome.

The overconsumption of a high-fat, high-calorie Western diets for long periods has dramatic effects on mammalian physiology, including weight gain and obesity, increased insulin resistance and diabetes risk, and increased risk of cancer and cardiovascular disease (1, 2). Reverting the physiological outcomes of HFD consumption can be mediated by food restriction, bariatric surgery, exercise, pharmacological intervention (*e.g.*, glucagon-like peptide-1 receptor agonists), or combinations of these (18). While exercise alone is not commonly effective in reverting these effects in humans, it does provide important improvements in insulin sensitivity and lipid metabolism, which may reduce cardiovascular event risk (19). In this study, we have examined the effect on the plasma proteome of exercise interventions applied either after the insult of the HFD (treatment) or at the same time as the deleterious HFD regimen (prevention). These data clearly show a strong effect of preventative exercise on ∼20% of the plasma proteins undergoing a significant response to a HFD alone. In contrast, using aerobic exercise as a treatment only had minor effects. It is interesting that only ∼25% of the HFD-responsive proteins had a significant response to exercise. These proteins were enriched for complement and coagulation-associated factors and acute phase proteins enriched for the Gene Ontology term response to stress (supplemental Fig. S1*C*). Many of these exercise responsive proteins are largely synthesized and secreted from the liver. As has been shown previously, exercise triggers myokine signaling to the liver to mediate regulation of liver function, which in turn effects the secretion of liver-derived proteins (20). One reason the preventative exercise regimen alone could provide superior beneficial changes for these proteins can be seen in the physiological data from these two models. These data showed that using exercise as a treatment had no significant effect on the body weight gain caused by the HFD (10) (supplemental Fig. S2), which was not unexpected and is widely observed in human clinical trials using exercise alone for weight loss. However, the prevention exercise regimen had a significant effect on body weight gain to an intermediate point between chow-fed animals and HFD-nonexercised animals (12) (supplemental Fig. S2). Therefore, prevention of animals reaching the maximum weight gain possible because of preventative exercise is likely a strong contributing factor to this effect. Preventative exercise may inhibit weight gain by the increased energy expenditure induced, from both the exercise intervention itself and its associated increase in spontaneous activity (12).

When HFDs are provided in an isocaloric fashion based on the energy consumption of mice fed a control chow diet, it is clear that most of the deleterious effects of a HFD are removed and animals may experience beneficial effects (5). For example, liver triglycerides are significantly lower in mice fed an isocaloric HFD, which may be correlated with decreased liver abundance of *de novo* lipogenesis enzymes (5). A comparison of the isocaloric HFD responsive plasma proteome with the data in this study from mice provided a HFD *ad libitum* shows that there are several interesting differences in plasma proteome profile. Top among these is the eightfold increase of the liver aldolase enzyme (ALDOB) in plasma from our *ad libitum* HFD-fed mice compared with their chow-fed controls. However, in isocaloric HFD-fed animals, the abundance of ALDOB is not significantly altered compared with that in controls (5). Liver aldolase is known to be increased in its plasma abundance after acute liver injury in humans and mice by acetaminophen (21), which is probably because of hepatic cell death/damage leading to the leak of abundant cytoplasmic enzymes into the blood. Similarly, a recent study has shown that high liver aldolase abundance in plasma is significantly associated with NAFLD in both humans and mice (22). Therefore, the overconsumption of a HFD and associated obesity probably leads to the increase in plasma ALDOB abundance that we observed here in mice through liver damage. Consistent with this concept, the plasma abundance of the alanine transaminase enzyme, which is characteristic of NAFLD, was significantly increased in HFD-fed mice (supplemental Fig. S2). While the change in ALDOB abundance we observed was not significantly corrected by the preventative exercise regimen, it was trending to be reduced by exercise with HIIT having the best corrective effect, which parallels a similar effect on alanine transaminase levels (supplemental Fig. S2).

CFD or adipsin is a largely adipocyte-derived protein (23), which we also observed to be differentially regulated by HFD overconsumption *versus* the isocaloric HFD dataset. Plasma CFD abundance was significantly decreased in HFD-fed animals by threefold, whereas mice fed an isocaloric diet high in either SFAs or PUFAs showed no significant change in CFD plasma abundance. Previous studies have shown that CFD is downregulated in rodent obesity models including HFD (24, 25) and that it is a protective molecule for pancreatic beta cells in diabetes rodent models and humans (26, 27). Our data show that exercise provided as a preventative regimen using either endurance or HIIT regimens was able to only partially revert the decrease in plasma CFD abundance because of HFD overconsumption. This may reflect its adipose tissue origin, and the potential inability of exercise-based signals such as myokines and metabolite changes to correct the reduced secretion of CFD.

Some proteins detected in this study were induced by any increase in fat consumption regardless of the composition such as apolipoprotein CII (APOC2), which activates lipoprotein lipase and facilitates fatty acid uptake from the circulation (28). However, the liver and adipose tissue can respond to fatty acids of different classes through the ligand-binding specificity of several transcription factors, including PPARA/G, SREBP1, and HNF4A (29). Our analysis shows that two highly abundant plasma proteins, Hp and α1-antitrypsin (SERPINA1E), had differential regulation of their plasma abundance in response to the saturation of fatty acids present in their diet. Hp was upregulated eightfold in the PUFA-rich isocaloric diet described previously (5) and by fourfold in our *ad libitum* PUFA-containing HFD dataset. The major function of Hp is to bind free hemoglobin in the blood, which can then be taken up by the liver for iron recycling (30). Hp is also an acute phase protein that has a myriad of other related functions, including antioxidant activity, antibacterial activity, and protease inhibition (30). In both humans and mice, Hp has been shown to increase in plasma abundance because of increased adiposity and increased adipose Hp secretion (31, 32, 33). Hp has also been shown in obese rats to increase by twofold when fed a diet that was 10% n-3 PUFA (34). This is in agreement with our observations of increased plasma Hp in mice fed a HFD *ad libitum*, when animals significantly increased their fat mass (supplemental Fig. S2). Hp is largely secreted by the liver, and increased plasma abundance of Hp can result from increased liver synthesis as is known to occur in response to toxins such as turpentine (35). Adipose tissue is also another source of Hp (25, 26, 27, 28, 30), whose secretion is increased by many factors including tumor necrosis factor-alpha and interleukin-6 (36). These cytokines are known to be increased in their abundance when animals are placed on a high PUFA diet (37). The transcription factor peroxisome proliferator–activated receptor gamma in adipocytes is known to be induced by PUFA binding (38, 39); however, treatment of adipocytes with a strong agonist like thiazolidinedione causes decreased Hp mRNA levels (40). This suggests that further work will be needed to delineate the tissue source of the Hp, the molecular mechanism leading to its increased plasma abundance, and the consequences of its increased plasma abundance on a HFD, which may include increased risk for cardiovascular disease (41).

In summary, this study provides a detailed comparison of the HFD response reflected in the plasma proteome of mice and the interaction between this diet and several exercise regimens used to correct those changes. Strengths of this study include the unbiased proteomic analysis methods used, which will sensitively detect significant changes in the more abundant plasma proteins. In addition, the C57BL/6J mouse model is a commonly used animal model for human disease including effects of changes in macronutrient dietary composition, and this study was complemented further through examination of two different exercise types, each as preventative or treatment regimens. Study limitations include that time points for analysis were fixed and few, and that the studies were undertaken in male mice only. This study has shown that a preventative endurance-based exercise regimen provided the best outcome to block HFD-induced changes. In addition, we were able to contrast the HFD response in our model of diet overconsumption and obesity with an isocaloric feeding model to identify several proteins specific for overconsumption of a HFD. This comparison also allowed us to delineate those changes regulated by SFAs or PUFAs in the diet. Future studies characterizing plasma protein turnover rates would allow us to delineate if these changes are due to a change in protein secretion from the tissues of origin or a change in the removal/degradation rate of these proteins from the plasma. We propose that the proteins identified here may be useful as markers of dietary composition and consumption history and as sources of information on the effectiveness of exercise-based interventions in humans.

## Data Availability

All the raw MS data and corresponding search outputs have been deposited to the ProteomeXchange Consortium (http://proteomecentral.proteomexchange.org) *via* the PRIDE partner repository with the dataset identifier PXD018561. Web-based visualizations for this dataset are available to the scientific community (larancelab.com/hfd-exercise).

## Conflict of interest

The authors declare no competing interests.
