# Supplementary material for: Blockade of High-Fat Diet Proteomic Phenotypes Using Exercise as Prevention or Treatment
Source: Mol Cell Proteomics. 2020 Dec 19;20:100027. doi: 10.1074/mcp.TIR120.002343 (PMC7950115; doi:10.1074/mcp.TIR120.002343)
Supplement: Supplemental Figures S1 and S2 [file mmc4.pdf]

Figure S1

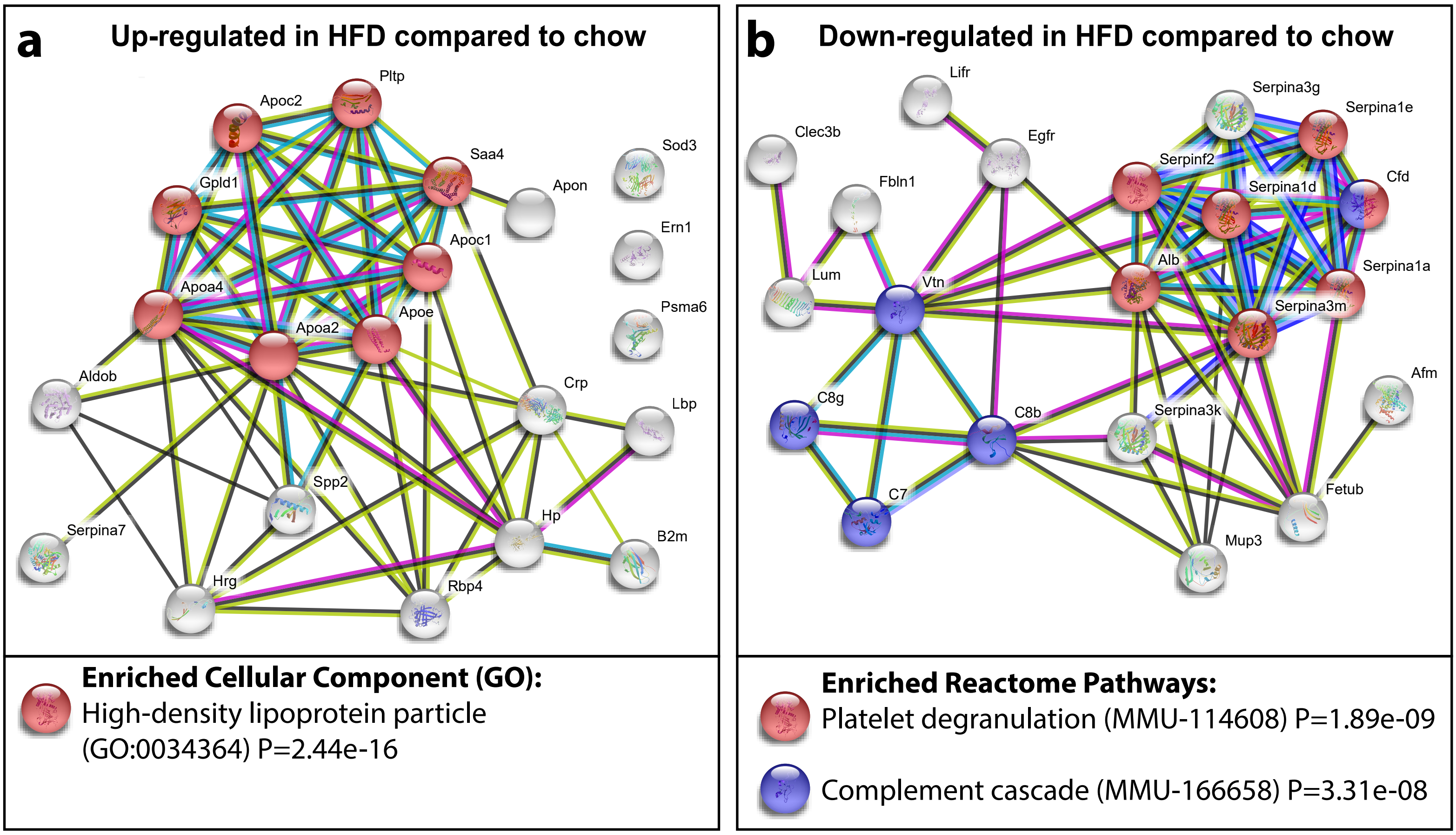

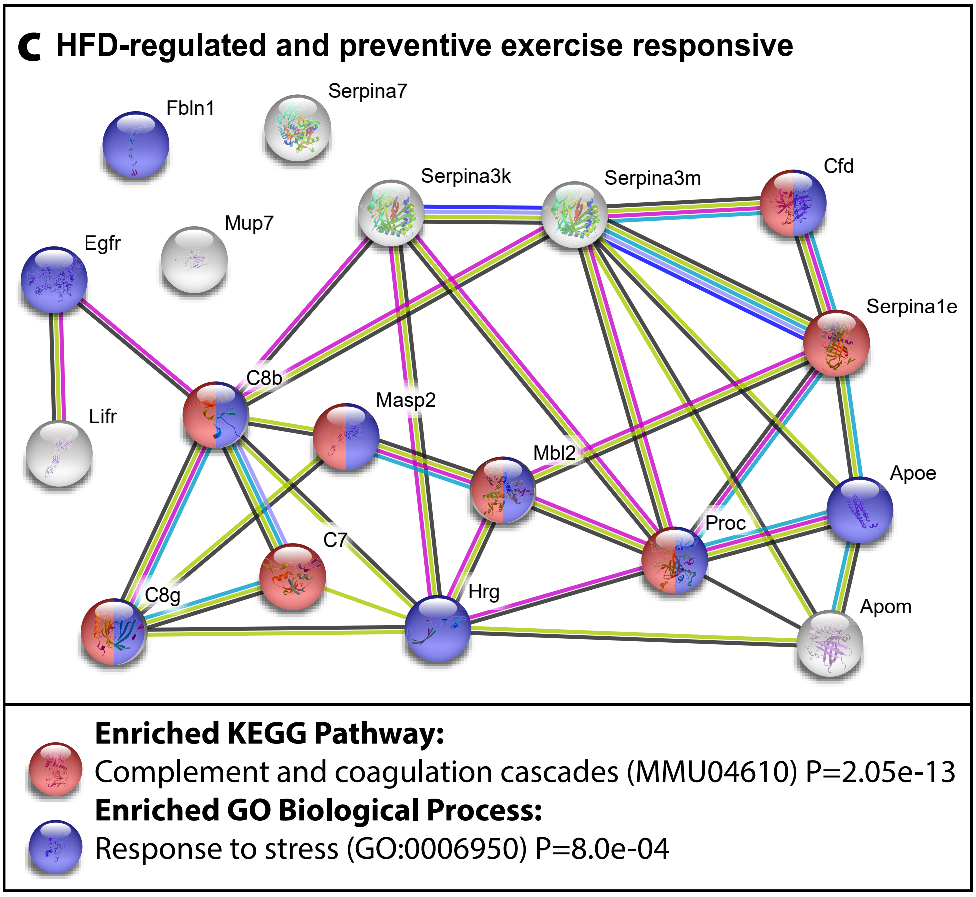

Figure S1. STRING Analysis of Significantly Regulated Proteins. Networks of interacting proteins within subsets of significantly regulated proteins were detected using the STRING database. Each protein is represented by a node and the lines between nodes indicate an interaction as defined in the legend. Gene set enrichment analysis has been used to identify significant and relevant protein interaction clusters, with common coloring of these nodes. (**a**) Proteins up-regulated in HFD compared to chow for both the treatment and preventative exercise regimes. (**b**) Proteins down-regulated in HFD compared to chow for both the treatment and preventative exercise regimes. (**c**) Proteins that were either up or down regulated in response to HFD and also showed a significant effect of exercise to block these changes in the preventative exercise regime.

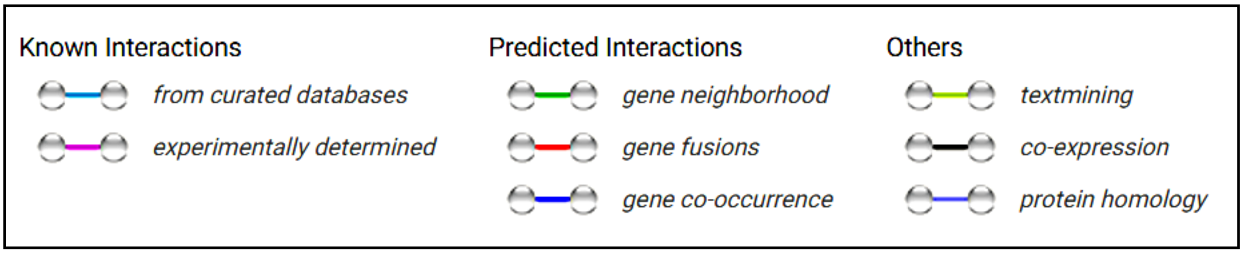

Figure S2

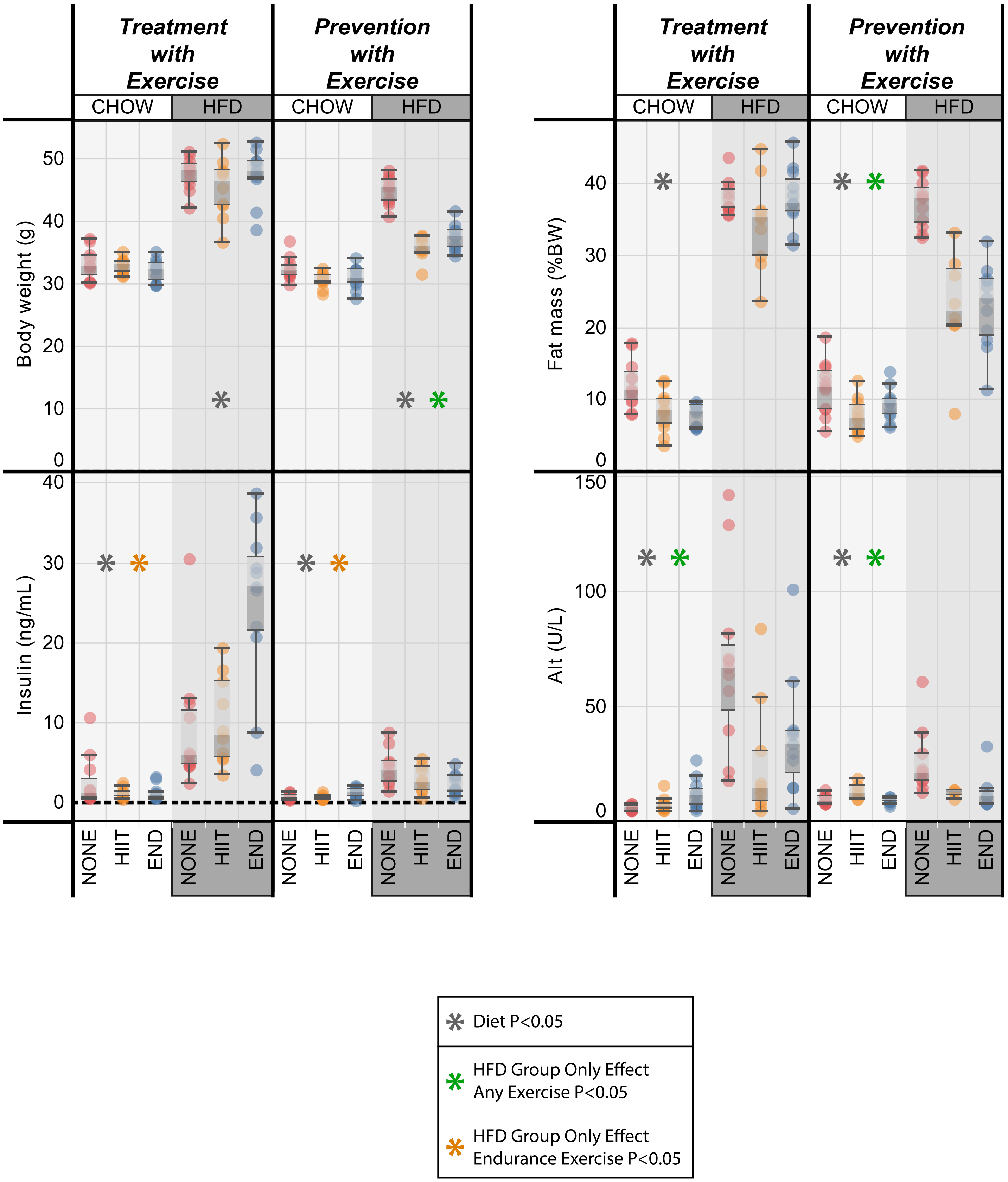

Figure S2. Mouse Model phenotypic and metabolic measures. Box and whisker plots for various physiological measurements from the mouse models used in this study as reported previously (10, 12), where each point represents data from an individual mouse. In general, HFD mice in the treatment and prevention regimes, were heavier, had greater % fat mass, higher circulating insulin and alanine transaminase (ALT) levels. Exercise regimes had the most notable effects in the preventative protocol, to attenuate the body weight gain, % fat mass and ALT levels. Asterisks placed at the top of the any plot represents statistical significance (P<0.05, or 5% FDR) across the treatment variables as indicated by the legend (n=12 per group).
